# Brain Bioelectric Responses to Short-Term Heart Rate Variability Biofeedback Training in Indian and Russian University Students Studying in the Russian Arctic

**DOI:** 10.3390/life15010011

**Published:** 2024-12-26

**Authors:** Denis Demin, Liliya Poskotinova

**Affiliations:** N. Laverov Federal Center for Integrated Arctic Research of the Ural Branch of the Russian Academy of Sciences, Arkhangelsk 163020, Russia

**Keywords:** biofeedback, heart rate variability, electroencephalography, Indian students, Russian students, Arctic

## Abstract

Heart rate variability biofeedback (HRV BF) training aids adaptation to new climatic, geographical, and social environments. Neurophysiological changes during the HRV BF in individuals from tropical regions studying in the Arctic are not well understood. The aim of this study was to research electroencephalographic (EEG) changes during a single short-term HRV BF session in Indian and Russian students studying in the Russian Arctic. The Indian (*n* = 40) and Russian (*n* = 40) healthy students (age 19–21 years) at a medical university in Arkhangelsk (64°33′ N 40°32′ E) were studied. HRV and EEG parameters were measured at baseline (5 min) and during a short-term HRV BF session (5 min) to increase the total power (TP, ms^2^) of the HRV spectrum. The baseline heart rate and stress index levels were significantly higher in the Indian students. During the HRV BF sessions, the sympathetic activity decreased more significantly in Russian students, while the alpha EEG activity significantly increased across all brain regions in both groups. In Indian students, there was a notable increase in theta and beta1 EEG spectral power in the frontal, central, and temporal regions. HRV BF training in Indian students was associated with a more pronounced activation of brain systems compared with Russian students.

## 1. Introduction

Psychological and physical adaptation to new climatic and social conditions during the first year of university is accompanied by considerable stress on the body’s compensatory and adaptive systems. For international students, additional stressors include adjusting to a new social environment, living conditions, language, community, and an unfamiliar country with its own culture and traditions [1]. In the Arctic environment, short-term adaptation to stress is characterised by increased anxiety, reduced mental and physical performance, borderline shifts in hormonal status, and activation of various brain structures [2]. The central nervous system and autonomic nervous system (ANS), which are actively involved in all adaptation and coping reactions, are among the most responsive human systems to changing environmental factors. Residents of the Arctic show a more complex structure of cortico-visceral connections, coherence in electroencephalographic (EEG) rhythms, and right-hemispheric dominance in brain functions compared with residents of southern regions [3]. Adaptation among international university students moving from tropical regions to the Arctic environment requires enhanced activity in various brain structures, significantly increasing the energy exchange, as reflected in the levels of direct current cerebral potentials (DC-potentials) [4,5]. The study of electrocardiography (ECG) data shows that young Arctic residents have a high level of centralisation of heart rate (HR) control and low activity of the parasympathetic section of the ANS [6]. Similar changes are observed in international students adapting to the Arctic’s educational and social environment [7]. Low ambient temperatures trigger activation of the sympathetic section of the ANS, aimed at increasing thermogenesis and maintaining adequate blood circulation in essential body areas [8]. However, while sympathetic responses drive catabolism and resource consumption, preserving parasympathetic resources is necessary to support the body’s anabolic processes. Therefore, effective parasympathetic regulation of cardiac activity is essential for achieving long-term adaptation in challenging environmental conditions.

Pharmacological management of these unfavourable functional conditions is often accompanied by side effects, making drug therapy an impractical option for enhancing the adaptive properties of human regulatory systems. Consequently, there is high demand for non-drug approaches that target systemic improvement in cognitive function and timely correction of psychogenic functional disorders [9]. Currently, the development of such tools is led by interface technologies (e.g., brain–computer interfaces and cardio–computer interfaces) as well as biofeedback (BF) technologies. In BF technologies, various biophysical characteristics of the human body are converted into information feedback signals, allowing individuals to learn voluntary regulation of various bodily functions [10]. Interfaces that harness the body’s endogenous rhythms demonstrate enhanced effectiveness because they leverage natural regulatory mechanisms and brain plasticity.

We have previously shown that HRV biofeedback training is an active cognitive process involving the search for relevant information, selection of essential features, and comparison of these features with each other, which indicates the active involvement of neurophysiological mechanisms in its formation [11]. Because of these characteristics, HRV BF procedures have been successfully used to reduce anxiety and psycho-emotional stress [12,13]. Additionally, HRV BF procedures may enhance cognitive activity and can train individuals to perform effectively under stress, thereby increasing their adaptive capacity [14,15]. It is also suggested that biofeedback training via heartbeat control can improve emotional learning and memory [16].

To date, the comparative aspects of brain activity and cardiovascular system response during the HRV BF training in both foreign and native people living in the Arctic environment have not been studied. This information is needed to develop corrective programmes aimed at reducing maladaptive states using the biofeedback approach in university students arriving from a tropical region to study in the Arctic. The purpose of this study was to investigate EEG changes during short-term HRV BF training in Indian and Russian students studying in the Russian Arctic. The scientific hypothesis of the study was that differences were expected in the functioning of the «brain–heart» system HRV BF training in Indian and Russian young people living in the Russian Arctic.

## 2. Materials and Methods

### 2.1. Subjects and Data Measurements

A cross-sectional non-randomised study was conducted with 80 clinically healthy male students, aged 19 to 21 years, from Northern State Medical University (Russian Federation, Arkhangelsk, 64°33′ N 40°32′ E). Two groups were formed: students who came from India and lived in Arkhangelsk for 1 year (Group I, *n* = 40) and Russian students of East Slavic ethnicity who were residents of Arkhangelsk (Group R, *n* = 40). Voluntary informed consent was obtained from all participants. The exclusion criteria were toxic substance use, a history of diagnosed brain injury, and neurological, psychiatric, or cardiovascular disorders. All studies were performed in October and November 2023. Before the study, the participants were verbally interviewed about their lifestyle, stress, and resting habits. There were no health disorders requiring observation by a general practitioner, cardiologist, neurologist, or other medical specialist, and there were no viral infections in the participants. The study period did not involve periods of examination sessions for students and was not accompanied by stressful social events. All students were examined under the same standardised conditions, at rest, at an air temperature of +22 + 24 °C, between 9 a.m. and 2 p.m. All participants had no training experience with the reported biofeedback method before the study, except one 5 min training session. Preliminary physical activity, consumption of tonic drinks, strong tea, coffee, alcohol, and smoking were also excluded.

### 2.2. Data Processing

The HRV parameters were recorded in a seated position using the «Varicard» device (Ramena Company, Ryazan, Russia). A reusable Ag/AgCl-coated medical steel sensor and connector terminal electrodes for extremities electrocardiography (ECG) were employed. These electrodes were used in conjunction with conductive gel. We measured the HR (bpm), total power (TP, ms^2^) of the HRV spectrum, standard deviation of NN intervals (SDNN, ms), root mean square of successive RR interval differences (RMSSD, ms), and the Baevsky stress index (SI, units) [17,18], reflecting the level of sympathetic effects on the heart rhythm. The SI was calculated by the formula SI = Amo50/2 × VAR × Mo, where Mo (ms) is the ECG R-to-R interval value dividing the ECG R-to-R interval series in half, VAR is the variation range between the minimum and maximum values in the ECG R-to-R interval series, and Amo50 (%) is the amplitude of the mode (the most frequent R–R intervals). The HRV parameters were recorded at rest (5 min) and once during the HRV biofeedback (HRV BF) session (5 min) [19].

**Figure 1:**
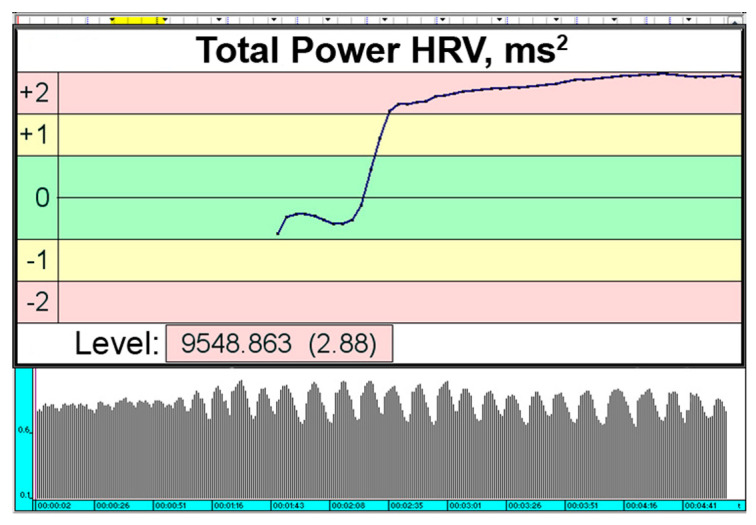
An example of a successful HRV BF session: raising the graph line and changing the amplitude and frequency of the ECG R-to-R intervals’ waves.

During the HRV BF session, the participants were instructed to maintain calmness, relax their muscles, and breathe with deep steady inhalations followed by smooth slow exhalations. The TP parameter was assumed to be the «controlled» parameter because the participant changed this parameter under their own visual control on the computer display the HRV BF session. The participants visually monitored the TP (the controlled parameter), which was displayed as a numerical value and graphical trend on a computer display. The TP was expected to increase in an effective HRV BF session [18,19] (Figure 1).

This indicator was updated every 4 s in the dynamics of the session according to the «sliding window» principle. Before starting the study, the participant was instructed that the change in the graphical TP trend on the display depended on the participant’s internal state, and the aim of the training was to increase the TP trend (Figure 1). The state reflecting the change of the selected parameter was formed by means of the strategy of «free search»—making positively mental images in combination with calm deep breathing with slow smooth exhalation and muscle relaxation [19].

EEG was recorded during the final 2 min of each study stage, in a seated position and state of calm wakefulness with eyes closed, using a Neuron-Spectrum-4/EPM electroencephalograph (NeuroSoft, Ivanovo, Russia). A monopolar setup with 16 standard leads was applied, following the international 10–20 system with ear reference electrodes. Standard Ag/AgCl-coated wire cup-shaped electrodes for EEG studies and standard conductive gel were used. For the EEG evaluation, 60 s, artifact-free recording segments were identified at each study stage. The EEG spectrum was analysed for theta (4.0–7.5 Hz), alpha (8.0–13.5 Hz), and beta1 (14.0–24.0 Hz) frequency bands. Quantitative assessment of the EEG spectrum in each frequency band was based on the absolute spectral power (µV^2^) values in the frontal (F3 F4), central (C3 C4), temporal (T3 T4), and occipital cerebral regions (O1 O2).

### 2.3. Statistical Analysis

Statistical analyses were performed using STATISTICA software v. 13.0 (StatSoft Inc., Tulsa, OK, USA). As the distribution of values in the samples did not follow the law of normal distribution (Shapiro–Wilk test), statistical processing was carried out using non-parametric methods. The quantitative HRV parameters are presented as medians with ranges corresponding to the 25th and 75th percentiles (lower and upper quartiles). Comparisons of quantitative variables between independent groups were conducted using the Kruskal–Wallis test, with a significance threshold of *p* < 0.05 for two-group comparisons. HRV parameters within each group, comparing HRV BF values to baseline values, were assessed using the Wilcoxon test (*p* < 0.05). EEG data are reported as a percentage increase in EEG power at HRV BF compared with baseline; however, significance levels on the graph represent comparisons between the absolute values of EEG spectral power during the HRV BF session and baseline withing each group, performed using the Wilcoxon test (*p* < 0.05). Additionally, comparisons between Groups I and R during the HRV BF session were conducted using the Mann–Whitney U test (*p* < 0.05).

## 3. Results

The baseline values of HR and SI were significantly different between the groups of Indian and Russian students (*p* = 0.031–0.038)—Table 1.

Higher baseline HR and SI values were observed in the group of Indian students. There were no statistically significant differences between groups in the TP of the HRV spectrum, SDNN, and RMSSD (*p* > 0.05). During the HRV BF session, the medians of the controlled TP parameter were statistically significantly increased in students from both groups compared with the baseline values (*p* < 0.001). The SDNN values also showed a significant increase in all students (*p* = 0.003–0.007), while the SI significantly decreased in all participants (*p* < 0.001). During the HRV BF stage, Group R students had significantly higher TP and lower SI indices than Group I students (*p* = 0.025–0.033). The HR and RMSSD did not change significantly in either group from baseline to HRV BF, although there was a tendency (*p* > 0.05) for these indicators to increase as the HRV BF session progressed.

**Figure 2:**
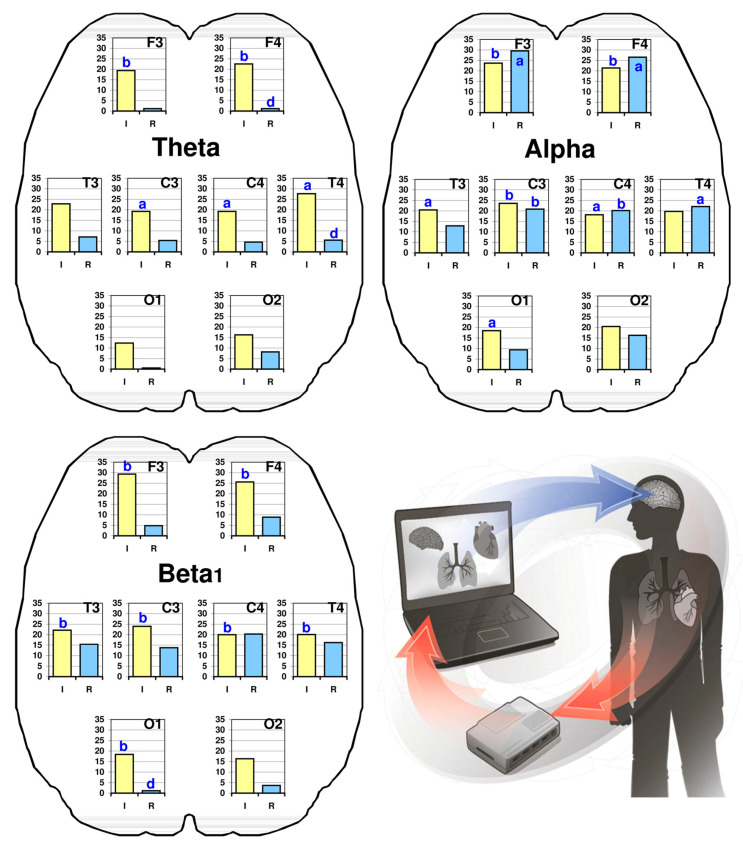
Percentage gains (in relation to the baseline value, in %) of the EEG spectral power in frequency EEG bands in the dynamics of HRV BF session in students from different regions. F3, F4, C3, C4, T3, T4, O1, O2—left and right frontal, central, temporal, and occipital EEG points. Yellow bars, Group I (Indians); blue bars, Group R (Russians). Statistically significant difference between baseline and after HRV BF session parameters in each group (Group I or Group R): ‘a’, *p* < 0.05; ‘b’, *p* < 0.01 and ‘d’, *p* < 0.05 between Groups I and R after HRV BF session. The coloured arrows schematically show the process of information exchange in real time between the dynamics of human physiological parameters and indicators of the device registering these parameters during the HRV BF.

In general, an increase in the spectral power of the main EEG frequency ranges from baseline to HRV BF stage was observed in participants from both groups (Figure 2). In men from Group I, significant increases in theta activity were noted across all brain regions, with the most pronounced increases in the frontal (F3 F4, *p* = 0.004–0.002), central (C3 C4, *p* = 0.015–0.028), and right temporal brain regions (T4, *p* = 0.033). Conversely, the increase in theta EEG activity power was statistically non-significant in Group R. A significant difference in percentage increases in EEG power between Groups I and R was found in the right frontal (F4, *p* = 0.024) and temporal regions (T4, *p* = 0.012).

Significant percentage increases in the EEG alpha-band spectral power during the HRV BF session were observed in participants from both groups. In Group I, the most substantial increases were found bilaterally in the frontal (F3 F4, *p* = 0.007–0.005), central (C3 C4, *p* = 0.005–0.032), and left temporal (T3, *p* = 0.031) and occipital (O1, *p* = 0.014) brain regions. In Group R, significant increases in alpha EEG activity were observed in the frontal (F3 F4, *p* = 0.014–0.035), central (C3 C4, *p* = 0.004–0.007) and right temporal (T4, *p* = 0.041) brain regions. In Group I, the spectral power of the EEG beta1-band significantly increased across all brain regions (*p* = 0.002–0.008), whereas in Group R, the increase in beta1 activity was only at a trend level (*p* > 0.05). Additionally, a significant difference in the percentage increase of EEG beta1-band spectral power between Groups I and R was observed in the left occipital region (O1; *p* = 0.018).

There was a statistically significant difference between the baseline and post HRV BF session parameters in each group (Group I or Group R): ‘a’, *p* < 0.05; ‘b’, *p* < 0.01; and ‘d’, *p* < 0.05, between Groups I and R after the HRV BF session. The coloured arrows schematically show the process of information exchange in real time between the dynamics of human physiological parameters and indicators of the device registering these parameters during the HRV BF.

## 4. Discussion

The HRV is directly controlled by the central nervous system and ANS and is one of the most important indicators of adaptation and adaptive processes in the human body [6]. The HRV method provides a non-invasive quantitative measurement of ANS activity to assess its adaptive capacity [17,18]. The ANS state of the students was assessed from baseline HRV indices. The SI of regulatory systems was used as a statistical characterisation of the dynamic series of ECG R-to-R intervals [18].

It is known that a lower SI value indicates greater activity of the parasympathetic branch of the ANS, while a higher SI value suggests increased sympathetic activity, greater centralisation of heart rhythm control, and an overall shift in autonomic homeostasis toward sympathetic dominance over parasympathetic activity. The RMSSD parameter was found to be less sensitive for assessing changes in vagal activity than the SI. As mentioned in the Methods section, the SI is the ratio of AMo (sympathetic activity parameter) and VAR, Mo (vagal activity parameters). With such a complex nonlinear rearrangement of autonomic heart rate regulation during biofeedback, SI can be considered the preferred index for tracking ANS changes. Additionally, the baseline SDNN and TP HRV values, which reflect the degree of parasympathetic influence on heart rhythm, were assessed [17]. The high baseline HR and SI values observed in the group of Indian students may indicate the predominance of sympathetic influences on cardiac activity. Previous studies have shown that the initial phases of social and educational adaptation in international students are characterised by dominant sympathetic regulation and increased centralisation of heart rhythm control, with stabilisation of these processes observed only by the third year of university [7]. Furthermore, migrants with incomplete mechanisms of climatic adaptation to the Arctic environment more frequently exhibit symptoms of sympathicotonia and arterial hypertension [20]. According to these authors, changes in peripheral haemodynamics are likely due to the activation of thermoregulation centres in response to cold skin receptor stimulation, which enhances the ergotropic activity of the sympathetic nervous system.

The vasomotor response of the cardiovascular system is mediated by sympathetic activation, which promotes peripheral vasoconstriction, thereby reducing heat loss limiting peripheral blood flow to body tissues [8]. In this study, we demonstrated that the students successfully followed the HRV BF procedure, increasing parasympathetic reserves even during a single short-term HRV BF session. The effectiveness of HRV BF was indicated by a decrease in the SI and an increase in the TP HRV, with participants achieving a state of general relaxation, calmness, mental comfort, and emotional balance. Compared to the SDNN, the TP parameter has minimal contributions from non-periodic (non-respiratory) ECG R-to-R interval waves, indicating that the BF effect primarily enhances the respiratory and baroreflex components of the HRV spectrum [15].

The use of breathing rhythm in biofeedback technologies is based on the concept that breathing significantly influences cortical brain activity through multiple sensory pathways [21,22], as well as the concept of cardiorespiratory synchrony, which suggests a close relationship between the cardiovascular and respiratory systems in regulating physiological status [23]. In respiratory biofeedback procedures, target parameters often include resonance or slow breathing [24,25]. Studies have shown that developing a stable skill in calm diaphragmatic breathing with slow exhalation helps normalise autonomic nervous system balance, improve respiratory function, and enhance overall well-being. Other studies have successfully applied biofeedback resonance breathing training in treating hypertension [26] and anxiety [27]. Acute stress has been shown to increase cerebral cortex excitability, an effect mediated by alpha2-adrenergic and glucocorticoid receptors [28]. Results from biofeedback training with diaphragmatic breathing, compared with a control group, demonstrate a significant decrease in cortisol levels and improved decision making under simulated stress [29]. The brain biopotential system, along with the cardiovascular and respiratory systems, has its own resonant frequencies, which coordinate optimal functioning. These systems exhibit phenomena of synchronisation and resonance and are highly sensitive to environmental factors [30,31].

The lack of significant changes in theta EEG activity in Russian students may indicate the relative stability of their subcortical diencephalic brain structures to changes in the ANS balance during the HRV BF procedure. Conversely, in Group I students, functional restructuring under HRV BF conditions led to pronounced activation of the hippocampal-cortical system, with a marked increase in theta EEG activity. Research has shown that even low-intensity but rhythmically organised signals can produce pronounced physiological effects, provided their frequency aligns with endogenous biorhythms, due to resonant mechanisms that alter biological states [32]. Indian university students who came to study in the Russian Arctic region exhibited elevated levels of cerebral direct current potentials across all brain regions, with central brain region values more than twice the normal levels; this indicated high energy consumption by the brain system [4]. The increase in the alpha activity spectral power across all EEG leads observed in this study reflects the dominance of the ascending activating influences from the reticular formation and thalamic structures on the neocortex [11].

The combined enhanced influence of thalamic and brainstem structures on the bioelectrogenesis of the cerebral cortex leads to an increase in alpha activity in the frontal and central brain areas [33]. It is known that individuals in a meditative and relaxed state often exhibit increased synchronisation of theta waves and slow alpha1 EEG waves in the frontal brain regions [34]. Studies have also shown that using breathing techniques, as opposed to spontaneous breathing, results in an increase in alpha EEG spectral power across the cortex [35]. In general, an increase in alpha EEG activity indicates an optimisation of cerebral cortical–subcortical relationships, contributing to a reduction in the sympathetic activity during the HRV BF training.

A significant increase in beta1 EEG activity in the frontal, central, and temporal regions of the brain in Indian students indicates extensive involvement of the sensorimotor cortex, medial basal brain, and brain regions that regulate the emotions [36] in developing an individual strategy to enhance biofeedback effectiveness as a cognitive activity. It is known that the mechanisms of EEG beta waves are influenced not only by the properties of cortical neurons but also by the thalamus, as well as intracortical and thalamocortical interactions. Additionally, there is evidence of beta1 rhythm generation within the 15–20 Hz range in the hippocampus, specifically in the cornu ammonis (CA1) region of the hippocampus [37]. Therefore, the functional specificity of the beta EEG range related to achieving a relaxation state is not necessarily expected.

When identifying key rhythmogenic structures in the central and autonomic nervous system, it is important to consider that specific parameters and resonant frequencies vary significantly between individuals but remain highly stable within each individual. This suggests that for more effective HRV BF, it is essential to determine a more specific and meaningful frequency range of endogenous oscillators tailored to each person. Ideally, this approach should involve the simultaneous use of complex oscillator frequencies that reflect the activity of different bodily systems—including the brain’s bioelectric activity, respiratory frequency parameters, and HRV.

India is known for its long-standing tradition of meditative practices, suggesting that the BF self-regulation strategy of Indian students may engage brain resources more fully, incorporating elements of meditation even during the cardio-BF control, where focusing on breathing alone is often sufficient for self-regulation. Additionally, the strain on cardiac activity in Indian students may contribute to increased brain activity during the HRV BF as part of their adaptation to Arctic conditions, reflecting a high «physiological cost» for adaptation. Overall, these findings underscore the importance of prolonged and multiple HRV BF sessions for students arriving in the North from Southern tropical regions, to expedite adaptation, particularly of the «brain–heart» system, to the Arctic environment.

Limitations of this study included using data from male participants only, because participation of female students was difficult due to religious reasons for the Indian citizens (placing EEG electrodes on the uncovered head). In addition, the baseline ANS tone of the participants was not taken into account in this article because the study of the «brain–heart» system in participants with different ANS tone requires a specific further study. Only time-based and geometric parameters of HRV estimation were used in this study, because spectral HRV parameters depend significantly on respiratory rate. A significant slowing of the respiratory rate (less than nine respiratory cycles per minute) during the HRV BF made the interpretation of HRV spectral indices incorrect from the perspective of the contribution of sympathetic and parasympathetic influences on heart rate [38,39]. Therefore, further study of a wider range of HRV indices, including nonlinear indices, during the HRV BF is a separate issue for future studies.

## 5. Conclusions

This survey of male students (aged 19–21) studying in the Russian Arctic revealed that Indian residents had significantly higher baseline HR and SI levels than Russian Arctic residents. During short-term HRV BF training, both groups showed a percentage increase in vagal activity according to the HRV data, with a more pronounced increase observed in Arctic residents. In students from both regions, EEG alpha band responses were notable across all brain regions, with a shift in the alpha EEG activity toward the anterior, central, and temporal brain regions; this reflects the combined enhanced influence of thalamic and brainstem structures on neocortical bioelectrogenesis. Minimal changes in EEG theta activity were seen in Russian students following HRV BF sessions, suggesting stability in subcortical diencephalic structures, whereas Indian students displayed the largest increases in theta waves in the frontal, central, and temporal regions. For Indian students, effective HRV BF training was characterised by a substantial increase in beta1 EEG activity, indicating broad involvement of the sensorimotor cortex and mediobasal brain structures. In Indian participants, self-regulation and enhanced vagal influence on heart rhythm were achieved through pronounced activation of cortico–visceral connections, with significant engagement of brain activity resources in the neocortex and diencephalic structures.

In Russian students, the increase in beta1 EEG spectral power was minimal in the central and temporal brain regions, possibly indicating reduced involvement of brain structures in heart rhythm control and a more autonomous cardiovascular function during the HRV BF training.

## Figures and Tables

**Table 1:** Heart rate variability parameters in the dynamics of heart rate variability biofeedback (HRV BF) session in Indian (I) and Russian (R) students ^a^.

Variables	Group	Baseline	HRV BF
HR, bpm	I	79.2 (73.8; 88.0)	83.9 (78.0; 92.4)
	R	69.1 (60.7; 78.0) ^d^	78.3 (69.2; 80.5)
SI, units	I	136.7 (88.0; 202.3)	60.9 (33.4; 112.2) ^c^
	R	90.8 (40.6; 168.2) ^d^	35.7 (19.2; 64.3) ^c,d^
TP, ms^2^	I	2103 (1249; 3058)	5390 (3093; 9123) ^c^
	R	2849 (1646; 5497)	7190 (5221; 9230) ^c,d^
SDNN, ms	I	49.2 (38.0; 58.3)	77.1 (58.6; 100.8) ^b^
	R	50.6 (41.2; 79.1)	86.9 (76.9; 137.7) ^b^
RMSSD, ms	I	36.6 (24.7; 49.1)	47.1 (27.3; 68.9)
	R	48.7 (34.3; 74.4)	49.1 (42.1; 71.0)

HR: heart rate; SI: stress index; TP: total power of HRV spectrum; SDNN: standard deviation of NN intervals; RMSSD: root mean square of successive RR interval differences. ^a^ Data are presented as median (lower and upper quartiles). Statistically significant difference between baseline and after HRV BF session parameters in each group: ^b^ *p* < 0.01; ^c^ *p* < 0.001, Wilcoxon test; ^d^ *p* < 0.05—between Groups I and R, Mann–Whitney U test.

## Data Availability

The original contributions presented in the study are included in the article, further inquiries can be directed to the corresponding authors.
